# Organ Donation: Behaviour and Beliefs in Sweden

**DOI:** 10.1177/23779608241257011

**Published:** 2024-05-24

**Authors:** Theres Södereld, Åsa Engström, Katarina Lindgren, Angelica Forsberg

**Affiliations:** 1 5185 Lulea University of Technology, Department of Health, Education and Technology, Division of Nursing and Medical Technology, Luleå, Sweden; 2 Intensive Care Unit 57, 69676 Sunderby Hospital, Luleå, Sweden

**Keywords:** Organ donation, behaviour, belief, transplantation, population, quantitative research

## Abstract

**Introduction:**

Barely one-fifth of people in Sweden have expressed their will regarding organ donation in the national Swedish Donor Registry, and the number of organ donations in Sweden remains low.

**Objective:**

The aim of this study was to map behaviour and beliefs regarding organ donation in Sweden.

**Methods:**

In a descriptive cross-sectional survey following a quantitative approach and 600 questionnaires were issued to randomly selected individuals across Sweden. Of them, 206 (36.3%) were completed. Data were analysed using descriptive statistics and presented as frequencies and percentages. Analytical statistical testing involved Pearson chi-square tests, Mann–Whitney *U* tests, and Kruskal–Wallis tests.

**Results:**

The results indicate a discrepancy between positive opinion about organ donation in Sweden and the number of people enrolled in the Swedish Donation Registry. The most common argument for not wanting to donate organs was the notion of being too old to. Although self-rated knowledge about organ donation was admittedly low, so was interest in interest in learning more about it. Younger patients more frequently wanted information than older patients did.

**Conclusion:**

Not wanting to donate organs due to age and/or illness may indicate a misconception. Making one's will known does not involve assessing one's health status or age but solely concerns the wish to do so. The findings thus raise an important question: How can people's interest in learning more about organ donation be induced in ethical ways?

## Introduction

Although the frequency of organ donation (OD) in Sweden has increased, the need for organs remains significant. When no alternative treatment is available for severely ill individuals, transplantation is a safe, well-established form of treatment that provides the opportunity to survive, improve one's health (Alhussein et al., 2018), and return to an active professional life (SOU, 2015:84, 2015). Of course, to enable donation and thereby transplantation, the public has to have a positive attitude towards donation and a high willingness to donate (Boonin, 2019). In Sweden, however, barely one-fifth of people have expressed their will regarding organ donation in the national Swedish Donor Registry (SDR; National Board of Health and Welfare, 2022). Moreover, compared with some other countries, the number of ODs in Sweden is low (International Registry of Organ Donation and Transplantation [IRODaT], 2022), and every year people awaiting organs die. Given those trends, mapping behaviour and beliefs regarding OD in Sweden is vital to determining ways to increase the frequency of OD by deceased individuals.

## Literature Review

The shortage of organs for donation is a significant national as well as worldwide problem (European Directorate for the Quality of Medicines & HealthCare [EDQM], 2022). In Sweden, an average of 90,000 people die every year, and of them, an estimated 200–270 people are potential organ or tissue donors (National Board of Health and Welfare, 2021). In 2021, 19.3 individuals per million in Sweden donated their organs upon dying, but compared with other countries, that number of donations is low (IRODaT, 2022). That same year, Spain and the United States, for instance, had approximately twice as many deceased ODs. Unfortunately, even in countries with higher rates of OD, people die while waiting for new organs (EDQM, 2022).

A positive attitude towards OD has long been shown to relate directly to a higher propensity to donate one's or a next of kin's organs (Dopelt et al., 2022; Feeley, 2007; Morgan & Miller, 2002). Worldwide, approaches regarding OD policies differ, with the two most common being the opt-in policy, which is based on explicit consent, and the opt-out policy, which is based on presumed consent (Boonin, 2019). However, in many countries with presumed consent, the next of kin has the right to veto and thus prohibit OD (Fabre, 2014), and Molina-Pérez et al. (2022) have also demonstrated that the family's involvement in decision-making about OD is important. Moreover, Costa-Font et al. (2021) and Rosenblum et al. (2012) have shown that the top reason that organs are not donated, under both the presumed and explicit consent systems, is refusal by the next of kin. In Sweden, presumed consent, or the opt-out policy, applies. Thus, to increase the number of ODs, Sweden removed the next of kin's ability to veto OD in July 2022. Although the sole option for OD in Sweden was donation after brain death, since 2020 OD following cardiac or circulatory death has been implemented in an attempt to increase the frequency of OD (National Board of Health and Welfare, 2022).

In Sweden, individuals have several ways to make their willingness to donate their organs known. In addition to notifying their next of kin or signing a donation card, people can register with the National Swedish Donor Registry. Murray et al. (2013) have shown that the number of ODs increases if the public registers their willingness to donate. Although investigations in Sweden have shown that approximately 80% of people have a positive attitude towards OD, the will of most deceased potential organ donors remains unknown (National Board of Health and Welfare, 2022). Rosenblum et al. (2012) have found that similar patterns occur in many registries around the world, such that fewer than 40% of the general public is registered to donate their organs despite high public support for OD.

Recent studies (e.g., Akbulut et al., 2020; El Hangouche et al., 2018; Stadlbauer et al., 2020) on the topic have described knowledge about and attitudes towards OD in different settings and contexts worldwide. Akbulut et al. (2020) and El Hangouche et al. (2018), for instance, found that their participants had little knowledge about the process of OD, while Stadlbauer et al. (2020) have reported that the chief influence on OD knowledge is family discussions, and that nearly half of their participants indicated the misconception that a person is alive even when declared brain-dead. El Hangouche et al. (2018) have also reported public concerns about medical errors and the trafficking of procured organs. Three of the world's major religions—Christianity, Islam, and Judaism—believe that altruistic acts such as OD are highly valuable (Rumsey et al., 2003) and that saving lives is a top priority; thus, none of those religions is opposed to OD (Bokek-Cohen et al., 2022). Nevertheless, subgroups within the mentioned religions can have different attitudes towards OD (Oliver et al., 2012; Randhawa & Neuberger, 2016). The Church of Sweden is Evangelical Lutheran, and a large share of Swedish citizens are members of the Church. At the same time, with approximately one-third of its population not identifying with any religion, Sweden remains one of the world's most secularised countries (Kasselstrand, 2015).

Many recent studies (e.g., Burns et al., 2017; Koons & Smeltzer, 2018; Nilsson et al., 2022) have investigated the context of transplantation, including people's experiences with being on the wait list for a new organ, which can cause great suffering, uncertainty, and insecurity about survival. However, studies mapping the general public's opinions, including their need for knowledge about OD, are scarce. Mapping the general public's behaviour and beliefs regarding OD in Sweden may contribute to enhancing knowledge about the OD process and refining legislation surrounding it. Mapping such behaviour and beliefs may also help with developing functional informational policies to improve the public's understanding of the importance of making their will known in the SDR and, in turn, potentially increasing the frequency of OD by deceased individuals. For those reasons, the aim of this study was to map behaviour and beliefs regarding OD in the Swedish population.

## Methods

### Design

This study followed a descriptive cross-sectional survey design with a quantitative mapping approach and adhered to STROBE guidelines (Cuschieri, 2019) for cross-sectional studies.

### Research Questions

Our study's research questions were as follows:

1. What attitudes does the Swedish public have towards OD?
2. What knowledge does the Swedish public have about OD in general?
3. Do attitudes towards OD differ according to gender, age, and/or level of education?
4. Does knowledge about OD differ according to gender, age, and/or level of education?

### Setting

The study was conducted at the national level in Sweden, a country with a population of approximately 10 million people, a mean age of 41.7 years, and a gender distribution that is basically equal (see Table 1). Activities involving transplantation and OD in Sweden are governed by three laws. The first is the Act on the Determination of Human Death (SFS, 1987, p. 269). According to Swedish legislation, a person is dead when all of their brain functions have been completely and irrevocably lost (i.e., total cerebral infarction). Second, the Act on Transplantation and Other Medical Purposes (SFS, 1995, p. 831) stipulates procedures for obtaining organs or other biological material from a living or deceased person for the treatment of disease or bodily injury in another person (i.e., transplantation) or other medical purposes. In July 2022, new amendments to the Transplant Act (1995, p. 831) came into force including, for example, the possibility of continuing intensive care interventions in the end stage of a patient's life to enable OD and removing of the next of kin's right to veto OD. Third and last, the Public Access and Secrecy Act (2009, p. 400) contains provisions on the handling of public documents by public authorities when registering, disclosing, and otherwise handling public documents. The law also contains provisions about confidentiality in public activities and the prohibition of disclosure of public documents.

**Table 1.** Participant Characteristics and Characteristics of the Swedish Population.

Characteristics	Study population	Swedish population
Sex n (%)		
Male	90 (43.6)	5,298,324 (50.3)
Female	116 (56.3)	5,223,232 (49.7)
Age m (SD)	52.9 (18.1)	41.7
Land of origin n (%)		
Sweden	180 (88.7)	8,375,882 (79,6)**
Other country	23 (11.3)	2,145,674 (20,4)**
Education n (%)		
Primary school	30 (14.9)	1,182,163 (11.0)**
High school	83 (41.1)	3,259,406 (31.0)**
College/University	85 (42.1)	1,773,148 (16.8)**
Other	4 (2.0)	No data *
Occupation n (%)		
Student	15 (7.6)	467,349 (4.4)
Employed	107 (54.0)	5,159,095 (49.0)**
On sick leave	3 (1.5)	219,632 (2.0)**
Unemployed	2 (1.0)	335,328 (6.4)***
Retired	64 (32.3)	724,106**
Other	7 (3.5)	No data*
Religion		
Atheist	47 (26.8)	No data *
Islam	7 (4.0)	No data *
Christianity	117 (66.8)	No data *
Catholic	4 (2.3)	No data *
Physical health		
Good physical health	186 (91.6)	No data*
Poor physical health	17 (8.4)	No data*
Mental health		
Good mental health	191 (97.0)	No data*
Poor mental health	6 (3.0)	No data*

Note: * no record of these characteristics in Swedish registers.

** *Statistics Sweden (2022)*

*** *Swedish Public Employment Service (* *2023). Percentage based on number of citicents in working age.*

### Data Collection

The researchers of this study sent out 600 questionnaires to randomly selected individuals across Sweden. The participants’ addresses were provided by the Swedish National Register of Personal Addresses (Statens Personadressregister [SPAR], 2019). The inclusion criteria for participating were being at least 18 years old and being registered living in Sweden. Of the 600 questionnaires, 25 never reached the intended participants due to unknown addresses, and eight participants declined to participate. Of the remaining 567 questionnaires distributed, 206 (36.3%) were successfully completed and returned following two reminders.

### Instrument

With support from a statistician, a questionnaire was constructed that was designed to map OD and beliefs about it in the Swedish population. Because the overall aim was to map an unexplored topic and because we had no intention to prove a hypothesis, the questionnarie was not statistically tested for reability or validity. Nevertheless, the researchers ensured that the items on the questionnaire were clearly worded and answered the research questions. The questionaire was also reviewed by a research group with knowledge about OD. The first part of the questionnaire addressed participants’ characteristics (see Table 1), whereas the second part consisted of items aimed at assessing participants’ behaviour and beliefs regarding OD (see Table 2). Some items were inspired by a survey conducted by the National Board of Health and Welfare (SOU, 2015, p. 84), and nominal, ordinal, and string variables were used in the questionnaire.

**Table 2.** Items in the Questionnaire and Number (n) and Proportions (%) of Participant Rtings.

Items	N (%)
1. Do you know that you can donate your organs to a severely ill person after your death?	
Yes n (%)	192 (93.2)
No	9 (4.4)
Missing	5 (2.4)
2. Do you feel that you have confidence in Swedish health care?	
To a high degree	150 (72.8)
To a low degree	54 (26.2)
Missing	2 (1.0)
3. Do you want to donate your organs after death?	
Positive to donate my organs	144 (69.9)
Undecided	48 (23.3)
Do not want to donate my organs	12 (5.8)
Missing	2 (1.0)
4. If you do not want to donate your organs, which is the most important argument? *	
My body should be intact after death	4 (10.0)
Low confidence in health care	2 (5.1)
I am probably too old to be an organ donor	20 (51.2)
My health situation means that I probably cannot become a donor	4 (10.2)
Personal reason	2 (5.1)
I do not want to think about death	6 (15.3)
Religious/spiritual/cultural reason	1 (2.5)
Missing	3 (1.7)
5. Have you registered your wishes in the SDR?	
Yes	69 (33.5)
No	135 (65.5)
Missing	2 (1.0)
6. Have you made your will known in some other way?	
Yes, by notifying next of kin	49 (23.8)
Yes, by signing a donor card	40 (19.4)
No	88 (42.7)
Yes, by both notifying next of kin and signing a donor card	10 (4.9)
Missing	19 (9.2)
7. Do you know how to register your will in the SDR?	
Yes	108 (52.4)
No	88 (42.7)
Missing	10 (4.9)
8. Would you like to receive more knowledge about how to register your will in the SDR?	
Yes	69 (33.5)
No	113 (54.9)
Missing	24 (11.7)
9. Do you feel that you have knowledge of how the information in the SDR is handled?	
To a high degree	30 (14.6)
To a low degree	167 (81.1)
Missing	9 (4.4)
10. Would you like to receive more knowledge about how the information in the SDR is handled?	
Yes	72 (35.0)
No	124 (60.2)
Missing	10 (4.9)
11. Do you feel that you have knowledge about who can become an organ donor?	
To a high degree	38 (18.4)
To a low degree	160 (77.7)
Missing	8 (3.9)
12. Would you like to receive more knowledge about who can become an organ donor?	
Yes	77 (37.4)
No	121 (58.7)
Missing	8 (3.9)
13. Do you feel you have knowledge about what it means to live while waiting for a new organ?	
To a high degree	41 (19.9)
To a low degree	157 (76.2)
Missing	8 (3.9)
14. Would you like to receive more knowledge about what it means to live while waiting for a new organ?	
Yes	40 (19.4)
No	156 (75.7)
Missing	10 (4.9)
15. Do you feel you have knowledge of what life is like for someone who has received a new organ?	
To a high degree	39 (18.9)
To a low degree	160 (77.7)
Missing	7 (3.4)
16. Would you like to receive more knowledge about what life is like for someone who has received a new organ?	
Yes	47 (22.8)
No	151 (73.3)
Missing	8 (3.9)
17. If you were seriously ill, would you be prepared to accept an organ?	
Yes	155 (75.2)
No	12 (5.8)
Undecided	30 (14.6)
Missing	9 (4.4)
18. Do you know the attitudes of relatives towards organ donation? *	
Partners	98 (58.0)
Parents	36 (21.3)
Grandparents	5 (3.0)
Close friends	13 (7.7)
Adult children	17 (10.0)
Missing	73 (35.4)
19. If you imagine a situation where someone close to you has died and that person's attitude towards organ donation is unknown, how would you approach the question of organ donation?	
I would allow organ donation	124 (60.2)
I would forbid organ donation	7 (3.4)
Undecided	67 (32.5)
Missing	8 (3.9)

*More than one answer was possible

### Ethical Considerations

The Regional Ethics Review Board approved our study (nr: 2019-00867). Together with the questionnaire, an informational letter describing our study's aim and context was provided to prospective participants. The letter emphasised that participating in the study was voluntary, that participants could withdraw from the study at any time, and that returning a completed questionnaire was considered to indicate consent to participate. A register including names, addresses, and coded questionnaires was established, and participants’ personal data and the numbered questionnaires were stored in separate locked spaces. Only the researchers, have had access to the data. Details about the register were also included in the informational letters sent to participants.

### Statistical Analysis

Statistical analyses were performed using SPSS version 26 (SPSS IBM, Armonk, NY, USA). Descriptive statistics were used to gather the sample's characteristics and response frequencies and are presented in this article as frequencies and percentages. Meanwhile, analytical statistics were used to determine significance. Questions with 4-point response scales were dichotomised into ordinal 2-point scales and are presented in the results as percentages. However, when analysing significance, the original 4-point ordinal scales were used. The responses “very good physical health” and “rather good physical health” were grouped as “good physical health”, and the responses “very poor physical health” and “rather poor physical health” were grouped as “poor physical health”. Questions with response options “to a very high degree” and “to a high degree” were grouped as “to a high degree”, and the responses “partly” and “not at all” were grouped as “to a low degree”. Participants’ ages were dichotomised into two groups—18–53-year-olds and 54–96-year-olds—according to the sample's mean age. Analytical statistics were gathered using Pearson chi-square, Mann–Whitney *U*, and Kruskal–Wallis tests, and a *p* value less than .05 indicated statistical significance. No analytical statistics were gathered for questions with string variables.

## Results

### Sample Characteristics

Table 1 presents the characteristics of the sample (*N *= 206), which consisted of 116 women (56.3%) and 90 men (43.6%) with a mean age of 52.9 years (range = 18–96 years). Most participants (*n *= 180, 88.7%) were born in Sweden, had completed high school (*n *= 83, 41.1%) or had a college or university education (*n *= 85, 42.1%), and were either employed (*n *= 107, 54.0%) or retired (*n *= 64, 32.3%). On the whole, the sample was thus slightly older, better educated, and more employed than the general Swedish population.

Table 2 shows the questionnaire items and the participants’ responses to them. The vast majority of participants (93.2%) indicated being aware of the possibility of donating organs after death. Participants also rated their confidence in Sweden's healthcare system, and the majority (78.0%) indicated a high degree of confidence. Most (69.9%) also responded positively about donating their organs after death, whereas approximately a quarter (23.3%) remained undecided. More than half of the participants (51.2%) who did not want to donate their organs indicated that they were probably too old to be organ donors. Despite positive responses to posthumously donating organs, barely a third of participants (33.5%) had registered their wishes in the SDR. Approximately a quarter (23.8%) reported having notified their next of kin, approximately a fifth (19.4%) had signed a donor card, but few (4.9%) had taken both actions. As for the rest, a considerable number of participants (42.7%) reported not having made their will known in any way.

Participants also rated the extent to which they had knowledge about OD. Less than half (42.7%) indicated having no knowledge about how to register in the SDR, and most (81.1%) did not know how information in the SDR is handled. Beyond that, more than three-quarters indicated having a low degree of knowledge about who is eligible to be an organ donor (77.7%), about the experience of waiting for an organ to be donated (76.2%), and about the experience of living with a donated organ (77.7%). When asked whether they wished to have more information or knowledge about the abovementioned items, most participants indicated that they would not.

Although majority of the participants (75.2%) indicated that they would consider accepting a donated organ should they need one, 14.6% remained undecided. Regarding their family members’ attitudes towards OD, knowing one's partner's attitude towards OD was the most common (58.0%), followed by knowing parents’ attitudes (21.3%). Of all participants, 60.2% would accept a donated organ if their next of kin desired it; however, nearly a third (32.5%) were undecided.

Table 3 shows differences among the participants based on gender, age, and level of education. Men indicated a significantly higher degree of confidence in Sweden's healthcare system than women did (men: 80.7% vs. women: 67.2%; *p *= .034). Meanwhile, more women than men reported having a high degree of knowledge about who is eligible to be an organ donor (men: 14.8% vs. women: 23.1%; *p *= .044), about experiences while awaiting for a donated organ (men: 14.8% vs. women: 25.7%; *p *< .001), and about living with a new organ (men: 13.8% vs. women: 24.5%; *p *= .009).

**Table 3.** Differences by sex, age, and Level of Education.

Items	Characteristic of participants					
Numbe scale)	Sex (n%) Man/Women	P	Education (n%) Lower/Higher	P	Age (n%) Younger/Older	P
*1*						
Yes	84(96.6)/105(94.6)	0.512^a^	107(93.9)/83(97.6)	0.059^a^	105(97.2)/86(93.5)	0.203^a^
No	3(3.4)/6(5.4)		7(6.1)/2 (2.4)		3(2.8)/6(6.5)	
*2*						
High degree	71(80.7)/76(67.2)	**0**.**034^c^**	88(75.2)/60(70.6)	0.784^b^	75(69.5)/74(77.9)	0.231^b^
Low degree	17(19.3)/37(32.7)		33(24.8)/25(29.4)		33(30.5)/21(22.1)	
*3*						
Willing	63(71.6)/79(69.9)	0.809^a^	76(65.0)/66(77.6*)*	0.075^a^	81 (75.2) / 62(66.7)	0.095^a^
Unwilling	6(6.8)/6(5.3)		10(8.5)/2(2.4)		3 (2.8) / 9 (8.6)	
Undecided	19(21.6)/28(24.8)		31(26.5)/17(20.0)		24 (22) / 24 (24.7)	
4*						
*5*						
Yes	29(33.0)/39(34.5)	0.817^a^	29(24.8)/40(47.1)	**<**.**001^a^**	38(35.2)/31(32.3)	0.702^a^
No	59(67.0)/74(65.5)		88(75.2)/45(52.9)		70(64.8)/64(67.7)	
*6*						
Next of kin	18(22.8)/30(28.3)	0.090^a^	24(22.9)/24(30.0)	0.659^a^	26(26.5)/(23(27.4)	0.873^a^
Donor card	14(17.7)/26(24.5)		20(19.0)/20(25.0)		24(23.5)/16(19.0)	
no	45(57.0)/42(39.6)		56(53.3)/31(38.8)		46(45.1)/41(47.6)	
Kin/card	2(2.5)/8(7.5)		5(4.8)/5(6.3)		5(4.9)/5(6.0)	
*7*						
yes	44(51.8)/62(56.9)	0.478^a^	53(47.3)/55(67.1)	**0**.**014^a^**	59(56.1)/49(55.2)	0.933^a^
no	41(48.2)/47(43.1)		59(52.7)/24(32.9)		47(43.9)/40(44.8)	
*8*						
yes	33(42.3)/35(34.3)	0.273^a^	43(40.6)/26(34.7)	0.369^a^	46(44.7)/22(29.1)	**0**.**024^a^**
no	45(57.7)/67(65.7)		63(59.4)/49(65.3)		57(55.3)/56(70.9)	
*9*						
High degree	7(28.7)/22(20.4)	0.479^c^	13(12.5)/15(17.8)	1.000^b^	10(9.6)/20(21.5)	**0**.**029^c^**
Low degree	80(71.3)/86(79.6)		95(87.5)/69(82.1)		94(90.4)/73(78.5)	
*10*						
yes	33(37.9)/38(35.5)	0.728^a^	37(33.3)/35(42.2)	**0**.**017^a^**	50(48.1)/21(23.1)	**<**.**001^a^**
no	54(62.1)/69(64.5)		74(66.7)/52(57.8)		54(51.9)/70(76.9)	
*11*						
High degree	13(14.8)/25(23.1)	**0**.**044^c^**	18(16.1)/20(23.8)	0.631^b^	18(17)/20(21.7)	0.662^b^
Low degree	75(85.2)/83(76.8)		91(83.9)/64(76.2)		88(83)/72(78.3)	
*12*						
yes	31(35.3)/45(41.3)	0.338^a^	41(36.0)/36(43.4)	0.419^a^	47(44.3)/30(32.6)	0.141^a^
no	56(64,7)/64(58.7)		73(64.0)/47(56.6)		58(55.7)/62(67.4)	
*13*						
High degree	13(14.8)/28(25.7)	**<**.**001^b^**	20(17.7)/21(23.8)	**0**.**015^b^**	13(12.3)/28(30.4)	**0**.**006^c^**
Low degree	75(85.2)/81(74.3)		53(82.3)/63(76.2)		93(87.7)/64(69.6)	
*14*						
yes	17(19.5)/23(21.5)	0.738^a^	23(20.5)/17(20.7)	0.531^a^	28(26.9)/12(13.2)	**0**.**018^a^**
no	70(80.5)/84(78.5)		89(79.5)/65(79.3)		76(73.1)/79(86.8)	
*15*						
High degree	12(13.8)/27(24.5)	**0**.**009^c^**	18(15.9)/20(23.8)	0.110^b^	14(13.2)/25(26.8)	**<**.**001^c^**
Low degree	75(86.2)/83(75.5)		95(84.1)/64(76.2)		92(86.8)/67(73.2)	
*16*						
yes	18(20.7)/28(25.7)	0.412^a^	27(23.9)/20(24.1)	0.646^a^	31(29.2)/16(17.5)	0.056^a^
no	69(79.3)/81(74.3)		86(76.1)/63(75.9)		75(70.8)/75(82.5)	
*17*						
yes	74(85.1)/79(73.1)	0.130^a^	84(75.0)/71(84.5)	**0**.**002^a^**	93(88.6)/61(67.0)	**<**.**001^a^**
no	4(4.6)/8(7.5)		9(8.0)/3(3.6)		1(0.9)/11(12.1)	
undecided	9(10.3)/21(19.4)		19(17.0)/10(11.9)		11(10.5)/19(20.9)	
18*						
*19*						
allow	61(70.1)/61(56.0)	0.119a	65(57.5)/59(70.2)	0.283^a^	70(66.0)/53(58.1)	0.503^a^
forbid	2(2.3)/5(4.6)		3(2.7)/4(4.8)		3(2.8)/4(4.5)	
undecided	24(27.6)/43(39.4)		45(39.8)/21(25.0)		33(31.1)/34(37.4)	

Note: Significant values are in bold

a) p-value from Chi-Square test.

b) *p-value from Kruskal-Wallis*

c) *p-value from Mann-Whitney U*

* Not analysed for statistical differences

A significant difference also emerged regarding level of education, such that more participants with a high level of education reported having registered their wishes in the SDR than participants with a low level of education (low: 24.8% vs. high: 47.1%; *p *< .001). Significantly more often in the group with a high level than a low level of education, participants reported having knowledge about how to register their will in the SDR (low: 47.3% vs. high: 67.1%; *p *= .014), wanted knowledge about how data in the SDR are handled (low: 33.3% vs. high: 42.2%; *p *= .017), and having a high degree of knowledge about the experience of waiting for an organ to be donated (low: 17.7% vs. high: 23.8%; *p *= .019). Significantly more participants with a high level of education also indicated being willing to accept a donated organ should they need one (low: 75.0% vs. high: 84.5%; *p *= .002).

By age, younger participants wanted knowledge on how to register their will in the SDR significantly more than older ones (younger: 44.7% vs. older: 29.1%; *p *= .024), while older ones reported having a high degree of knowledge about how information is handled in the SDR significantly more than younger ones (younger: 9.6% vs. older: 21.5%; *p *= .029). Younger participants also indicated wanting knowledge about how information in the SDR is handled more often than older ones (younger: 48.1% vs. older: 23.1%; *p *< .001). When participants were asked about their knowledge about waiting for a donated organ, older respondents indicated having a high degree of knowledge more often than younger ones (younger: 12.3% vs. older: 30.1%; *p *= .06), while younger ones wanted more knowledge (younger: 26.9% vs. older: 13.0%; *p *= .018). Older participants indicated having a high degree of knowledge about living with a donated organ more often than younger ones (younger: 13.2% vs. older: 26.9%; *p *< .001); however, when asked if they would consider accepting a donated organ for themselves, more of them replied in the affirmative than the older participants (younger: 88.6% vs. older: 67.0%; *p *< .001).

## Discussion

The aim of this study was to map behaviour and beliefs related to OD in the Swedish population. In general, people's attitudes towards OD are consistently favourable (Najafi & Manzari, 2017; Ríos et al., 2019). This study, however, revealed a discrepancy between participants’ wishes to donate their organs (69.9%) and having registered those wishes in the SDR (33.5%). Previous studies (Mohs & Hübner, 2013; Wakefield et al., 2010) have shown that the shortage of organs for transplantation often stems from the poor translation of positive attitudes toward OD into registration as an organ donor. This study also showed that more participants with a high (vs. low) level of education had registered their will to donate in the SDR. Li et al. (2016) found similar results in an immigrant population, namely that a higher level of education was associated with a greater likelihood to register as a donor in an OD registry. In this study, another association emerged between an attitude against OD and not wanting to think about death (15.3%). OD may indeed be difficult to discuss because reminds people of their own mortality or can seem too abstract to relate to. Further research on how to increase public knowledge about OD is therefore needed.

Among this study's participants, the most common argument (51.2%) for not wanting to donate one's organs was a sense of being too old to have organs that are appropriate for transplantation. That reasoning, found in other studies as well (Abecassis et al., 2012; Loughery et al., 2018), indicates a lack of public awareness about OD and the belief that chronic disease and/or old age make OD impossible or that respondents distrust the allocation system. However, the findings of this study suggest that trust in healthcare in Sweden is rather high. Even so, Aijing et al. (2016) found that OD-related behaviour was influenced by mistrust in the process of how death is declared and the idea that a patient who agreed to donate their organs might receive insufficient care. Important parts of pro-OD work thus seem to be providing the public with correct information and advocating donation.

While the effects of campaigns in Sweden have not only been limited but rapidly dwindled as well (SOU, 2015, p. 84), other interventions have been tried (e.g., in primary care settings) in other countries with mixed results (Jones et al., 2017; Li et al., 2022). Jones et al. (2017) reviewed interventions in primary care settings and determined that ones that took a more active approach had a higher rate of success. More recently, Li et al. (2022) developed an intervention in which reception staff in primary care offices invited patients to register their will to donate in the waiting room; however, that intervention had no effect on rates of registration.

In this study, participants’ self-rated knowledge about OD was low as well, which reflects the results of past studies (e.g., Akbulut et al., 2022; Fan et al., 2022; Flynn et al., 2023). Interest in learning more about OD was similarly low. Despite those trends, the importance of knowledge about OD in decision-making about OD has been described by Krupic (2020), and previous studies (Tarzi et al., 2020; Umair et al., 2021; Wakefield et al., 2010) have shown that increased knowledge about OD correlates with more positive attitudes towards OD. Added to that, Tarzi et al. (2020) has shown that more knowledge about the concept of death was connected to more positive attitudes towards OD. By the same token, both they and El Hangouche et al. (2018) reported a general lack of knowledge about legislation concerning OD and transplantation and about practical issues with expressing one's will to be an organ donor.

Although Ríos et al. (2019) have argued that campaigns to improve OD rates should focus on processes involved in coordinating transplantation, not raising awareness of OD or improving the public's knowledge about and attitudes towards it, more than half of the participants (52.4%) in our study lacked knowledge about how to register their wishes in the SDR. On that point, multiple studies (McGlade & Pierscionek, 2013; Rodrigue et al., 2009) have also suggested that repeat exposure to advantageous information about OD is important and provides an opportunity for people to register immediately (Deedat et al., 2013). Other studies (Kessler & Roth, 2012; Li et al., 2013; Stoler et al., 2016) have described another policy approach, wherein people who have opted into OD are prioritised on the wait list should they need an organ transplant. Under those priority rule systems, rates of OD have been shown to increase.

In this study, younger participants lacked knowledge about OD and wanted information about OD more than older participants did. At schools worldwide, educational programmes and interventions about OD in different grades have been piloted with positive outcomes (Cárdenas et al., 2010; Milaniak et al., 2010; Piccoli et al., 2006; Siebelink et al., 2017). More recently, Almela-Baeza et al. (2022) tested another approach in which adolescents participated in creating a brief educational movie about OD and observed positive results in terms of knowledge about OD. Added to that, Ríos et al. (2022) tested the effectiveness of an opinion questionnaire on attitudes about posthumous OD among adolescents; however, completing the questionnaire had no positive effect on adolescents’ attitudes. Other researchers (Arisal & Atalar, 2020; Hajjar et al., 2016; Tarzi et al., 2020) have shown that TV, the internet, and other media are the top sources of information about OD and transplantation; however, Morgan et al. (2005) found that most negative opinions about OD related to information obtained from mass media. By contrast, Weng et al. (2021) found that, in most cases, positive opinions about OD were credited to personal beliefs and values. Healthcare professionals were the source of information in very few cases despite being more accurate sources (Hajjar et al., 2016;). In Sweden, healthcare professionals are not responsible for education about OD other than in nursing education at the university level. Inviting healthcare professionals to talk about OD in schools, with age-appropriate materials, may be a way to enhance OD-oriented behaviour in the general public.

In this study, most participants (60.2%) would allow for their next of kin to be an organ donor, although approximately one-third (32.5%) remained undecided. Those rates align with Şenyuva's (2022) results found in a Turkish context. When the questionnaire of this study was conducted, Sweden's approach to OD was presumed consent, and next of kin had the right to veto; however, the next-of-kin veto option was recently removed (SOU, 2015, p. 84). It is thus the next of kin's responsibility to interpret the wishes of their loved one if their wish is unknown. In Liu et al.'s (2021) study, participants were confident that they could interpret the wishes of their next of kin, even if they were unaware of their wishes prior to death. Such decision-making shifted depending on whether the decision was being made for themselves or for others. Participants who were willing to donate their own organs could refuse to let their next of kin donate. Also in that vein, Shepherd et al. (2023) found that the next of kin's negative attitudes towards OD was more likely to affect the approval of OD than OD's perceived benefits. To shift those trends, Shemie et al. (2017) have stressed the importance of having compassionate, supportive discussions with family members so that OD-related decisions can be informed and donors’ wishes respected.

### Strengths and Limitations

This study has several limitations. First, the questionnaire, which the researchers of this study designed, was inspired by an existing survey and the researchers’ knowledge as former responsible nurses and anaesthesiologists involved with OD. The questionnaire was not tested for validity or reliability; therefore, generalisability in statistical terms is not applicable. Instead of pursuing generalisability, the researchers intended to map OD-related behaviour and beliefs in Sweden following a primarily descriptive approach. To that end, the context specific to OD in Sweden and the characteristics of participants have been described well and allow the transfer of our results to other similar contexts. Moreover, having discussed the items in the questionnaire thoroughly, the researchers concluded that they would yield responses that would help them to achieve the study's aim. The questionnaire thus has face-validity.

Second, the response rate was low (36.3%), which is a significant limitation. Moreover, only self-report data were collected, which could have influenced the results. However, the sample in the study was randomly selected from the general Swedish population via SPAR and then mirrored against Swedish population data (see Table 1). As a result, deviations between the sample population and the Swedish population can be determined.

Third, the researchers chose to dichotomise the questionnaire items with ordinal 4-point response scales into nominal 2-point scales when presented in the results, which can be both a weakness and a strength. Dichotomising ordinal scales risks losing nuance. Although the researchers dichotomised the scales with the intention of having fewer and larger groups and to facilitate the understanding of results in clinical practice, while analysing significance Kruskal–Wallis and Mann–Whitney *U* tests were used for the original 4-point scales.

## Conclusion

This study revealed a discrepancy between participants’ stated wish to donate their organs and their having registered that wish in the SDR. The most common argument for not wanting to donate was the misconception of probably being too old to do so. Making one's will known does not entail an assessment of one's own health status or age but only the declaration of one's wishes. Moreover, the findings suggest that self-described knowledge of OD is low in Sweden and, surprisingly, so is interest in learning more about it. The findings thus raise an important question: How can people's interest in learning more about OD be induced in ethical ways, both in Sweden and beyond?

## Implications for Practice

Information about OD and transplantation must be enhanced, and it might be helpful to make providing such information compulsory. As a society, Sweden needs to dare to talk about death. This study has shown that younger participants were more likely to want information about OD-related issues than older ones. Given that finding, healthcare professionals may engage in repeat activities at schools to enhance OD-oriented behaviour using age-appropriate materials. Meanwhile, older adults need to receive customised messaging, including that age and illness do not disqualify them from making their will known in the SDR, which can be done via primary care practitioners and comes with the opportunity to register in the SDR at the same time. Beyond that, the discrepancy between the participants’ positive opinions about OD and the current percentage of Sweden's population who has registered their wish to donate their organs in the SDR should be further examined. Research on ways to increase public knowledge about OD is also suggested, as is research that explores critical care nurses’ and anaesthesiologists’ experiences with promoting OD and challenging factors during OD.
